# Ten ways to get a grip on resident co-production within medical education change

**DOI:** 10.36834/cmej.67919

**Published:** 2020-03-16

**Authors:** Samantha Buttemer, Jena Hall, Liora Berger, Kristen Weersink, J. Damon Dagnone

**Affiliations:** 1 Queen’s University, Ontario, Canada

## Abstract

The Royal College of Physicians and Surgeons of Canada (RCPSC) is transforming its national approach to postgraduate medical education by transitioning all specialty programs to competency based medical education (CBME) curriculums over a seven-year period. Queen’s University, with special permission from the RCPSC, launched CBME curricula for all incoming residents across its 29 specialty programs in July 2017. Resident engagement, empowerment, and co-production through this transition has been instrumental in successful implementation of CBME at Queen’s University. This article aims to use our own experience at Queen’s in the context of current literature and rooted in change leadership theory, to provide a guide for educators, learners, and institutions on how to leverage the interest and enthusiasm of trainees in the transition to CBME in postgraduate training. The following ten tips provides a model for avoiding the “black ice” type pitfalls that can arise with learner involvement and ensure a smoother transition for other institutions moving forward with CBME implementation.

## Abstract (fr)

Le Collège royal des médecins et chirurgiens du Canada (CRMCC) s’emploie à transformer son approche nationale à la formation médicale postdoctorale en effectuant une transition, répartie sur une période de sept ans, de tous les programmes spécialisés vers des programmes de formation médicale axéesur les compétences (FMAC). En juillet 2017, l’Université Queen, avec une permission spéciale du CRMCC, a lancé des cursus de FMAC pour tous les nouveaux résidents de ses 29 programmes spécialisés. La participation, la capacité d’agiret la coproduction par lesrésidents pendant cette transition ont contribué à la mise en œuvre réussie de la FMAC à l’Université Queen. Le présent article vise à utiliser notre propre expérience à l’Université Queen dans le contexte de la littérature actuelle et est ancrésur la théorie du leadership en matière de changement, pour procurer un guide aux éducateurs, aux apprenants et aux établissements sur la manière de tirer parti de l’intérêt et de l’enthousiasme des apprenantsdans la transition vers la FMAC dans la formation postdoctorale. Les dix conseils suivants proposent un modèle pour éviter les écueils du type « glace noire » qui peuvent survenir avec la participation de l’apprenant et s’assurer une transition plus harmonieuse pour les autres établissements qui vont de l’avant avec la mise en œuvre de la FMFC.

## Introduction

Transformative change in postgraduate medical education is upon all of us in Canada. The Competency by Design project, introduced by the Royal College of Physicians & Surgeons of Canada (RCPSC) in 2014 has launched medical educators, trainees, and leaders into implementation of competency-based medical education (CBME) across all postgraduate specialty training programs over the coming decade.^1^ As a result, there are many challenges ahead as we collectively navigate the design, launch, and ongoing implementation of CBME at our own institutions.

To ensure success, creating a leadership environment with resident trainees that values co-production, increasing empowerment, and end-user design is essential.^2^ Co-configured learning adaptive to the needs of the trainee is critical for any outcomes-based education model, including CBME, and mutual learning is needed between both trainer and trainee.^3^ CBME is a complex intervention requiring active input from all involved, and implementation requires a coalition spanning across clinicians, educators, residents, and administrative/support staff to be successful.^4^

**Figure 1:**
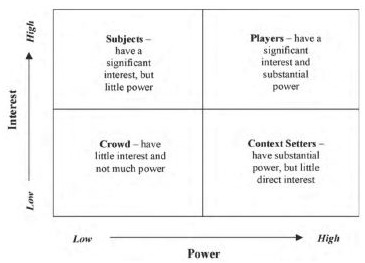
Stakeholder engagement grid, from Bryson et al

As active participants in their education, residents have substantial ongoing responsibilities in implementation of CBME. Stakeholders can be conceptualized as members of one of four groups (Figure 1). Often, most of the effort is placed on the high-power/high-interest individuals,^5^^,^^6^ despite the reality that those with less power but high interest, such as residents, face the day-to-day impacts of the change process. Involving these groups in the vision and implementation of change promotes the understanding of ground-level implementation issues and creates a smoother transition.

Based on our collective experiences of launching all 29 specialty programs into CBME curricula at Queen’s University in 2017, we describe 10 ways to get a grip on navigating the “black ice” of resident involvement in the implementation of CBME at the institutional level.

### 1. Create an explicit shared vision with a CBME Resident Committee

Creating a common vision amongst stakeholders buildsa sense of shared purpose and community, while providing a source of intrinsic motivation to all those involved. Intrinsic motivation is crucial for ensuring ongoing engagement.^7^ Bringing all stakeholders together in the early stages helps identify the overlapping themes from individual goals to create a unified vision. This approach fosters co-production from the first stages of change, creating a climateof shared values, ownership and pride for the change process and outcome.

To achieve this vision, the most important initial step is the establishment of a CBME resident committee. Ideally, this committee would be co-chaired by both a resident and faculty lead with membership from other residency program leads, a few early career faculty members, a medical student representative, and an institutional technology lead. Thematic issues, new challenges, and strategic solutions could then be brought to the higher level decanal leaders by the chairs of this committee so that residents have an impactful voice in the development of the institutional vision and strategy.

### 2.Leverage central support structures

Taking an institution wide approach to transition to CBME allowed us to leverage central support structures, a key in ensuring success^.^^8^^,^^9^ This approach allowed for an equitable governance structure in which all stakeholders were accountable to one another. The entire institution was navigating the creation and transition to new residency curricula together. Although the CBME committees were created following a traditional hierarchical accountability structure in which the various subcommittees report upwards to the executive, programs also maintained horizontal accountability to each other as they worked through challenges and shared experiences with one another. The approach taken contributes to an institutional environment of shared and transparent decision making.

Ongoing communication channels were established between key leaders and on-the-ground faculty, residents, department chairs, hospital administrators and other stakeholder groups to give insight to members of the CBME executive team in formal and informal ways. Constant check-ins, through one-on-one conversations, planned meetings, and program leader workshops attended by both residents and staff, provided insight into the evolving institutional needs.

### 3. Identify, invest in, and empower champions of change

Change brings with it new projects requiring time and work, and cannot be accomplished without champions ofchange to lead the process.^10^^,^^11^ Each program at our institution identified new champions for each of our 29 programs: a CBME Faculty Program Lead (FPL) and at least one Resident Program Lead (RPL), who partnered with existing program directors to undertake CBME implementation. The RPL role is intended to be filled longitudinally by the same resident over two or more years. It is not tied to any other role, such as chief resident or union representative, but rather is filled by a resident with special interest in medical education. By explicitly placing value on these new champion roles, through the central academic funding formula (including new funding for FPL and RPL events, role descriptions, and clear deliverables), these new positions became highly valued and are now built into the leadership structures across all programs.

Workshops for RPLs on change leadership further developed the knowledge and skills to become an active participant in their own program’s ongoing implementation and the faculty CBME workshops. Program directors were encouraged to support RPLs by allowing time away from clinical activities to attend CBME workshops, creating space for PGME-wide co-production.

### 4.Engage stakeholders throughout

The systems-based approach to the CBME governance structure reflects the value of key stakeholders within the greater community moving through the change, and the need for involvement of all stakeholders, including trainees. Therefore, our institution placed such importance on FPLs and RPLs. These champions hold social capital amongst their colleagues allowing for easier faculty and resident development and ongoing clear, non-threatening, and open communication channels through the change process. Practically, this meant that each lead works both to design CBME curricular requirements, and act as a voice for the body of stakeholders they represent. As well, the leads are encouraged to meet regularly to ensure the resident voice is heard by faculty throughout implementation in all programs. While no one individual can entirely express the interests of those within their group, they can still act as a voice to amplify the concerns of those around them. This ensures the space for issues to be brought to the CBME Executive Committee, keeping it accessible for all levels of stakeholders.

### 5. Provide a technical infrastructure that grows and adapts with the project

Technical infrastructure is key to supporting the needs of those involved in the CBME transition.^12^ A user-friendly adaptable information technology (IT) platform to collect residents’ performance information is integral to CBME. With increased emphasis on direct and indirect observation, the IT assessment tools provided to the assessors must support concrete, timely, and actionable feedback that is recorded. A functional system is necessary for supporting the ongoing practice of frequent evaluation, and any technical barriers may cause early disengagement from the task.^13^

At Queen’s, the IT team works directly with the CBME resident subcommittee, implementing changes to optimize both user-friendliness and learning from the assessment platform. Continuing multiple open channels of communication using an online ticket system, subcommittee meetings, leadership updates, workshop forums, and individual program meetings has been effective. The IT team’s willingness to respond to resident needs and quickly adapt the functionality of the system motivated residents to remain actively engaged in improving the platform and CBME more broadly.

### 6.Get creative with communications

Knowledge translation has become a hot topic in academia, especially with the advent of social media. Non-traditional avenues of communication such as blogs, podcasting, and twitter help promote ideas and concepts.^14^ Targeted communication through multiple avenues increases the chance of the message being heard.

As a committee, we created visuals, such as infographics and posters, and spread them across the hospitals and common resident spaces. CBME lanyards, pins, and T-shirts were used as a visual conversation starter and symbolized CBME interest to members of the health care community. Presentations were also given by RPLs to each of their respective specialties, and socials were organized at local pubs to create an informal venue for open communication.

External to Queen’s, residents from the CBME resident subcommittee contributed to education blogs to reach the online community and participated in events and interviews to increase visibility and subsequently resident awareness.^15^^,^^16^ RPLs have presented and coordinated workshops at various national and international conferences with support from the university, sharing experiences with resident co-production widely.

### 7. Capitalize on early wins to build momentum

To keep people engaged in the change process, emphasis is needed on early wins.^9^ Within our subcommittee, RPLs took on roles that were best suited to their interests and abilities instead of being delegated tasks. Monthly meetings enabled these efforts to be celebrated in-person, with a recurring agenda item for celebrating presentations, awards and efforts made by the group and individuals. Inspired by self-determination theory as a framework for understanding intrinsic motivation, we chose to deliberately celebrate individual successes as recognition of competence and autonomy in creating change and lauded the group successes as a way of creating relatedness and a sense of community.^8^

As the Queen’s CBME resident subcommittee gained momentum, the activities grew in number, and the wider group of stakeholders became increasingly aware of the value of our subcommittee. Once our committee contributions were perceived to be valuable, support for resident engagement in the implementation process became prioritized and is now consistently sought and valued across programs.

### 8. Anticipate and mitigate the change dip and embrace iterative processes

The journey of change from initial conception to completed implementation is not linear in its upward trajectory, but instead has a predictable “dip” in uptake. Individuals’ experiences through this dip can be explained by the following processes: denial, resistance, disorientation, experimentation, and commitment.^17^ Focusing on a growth mindset and reframing failed interventions and difficult situations as opportunities to rework our approach moved us towards the change we hoped to see occur.^17^^,^^18^

Rather than have a rigid mandate, the resident subcommittee was encouraged to embrace emergent strategies when newly identified challenges arose. To identify and address these causes we adopted the SCARF framework as a way to understand resident and staff resistance to change.^19^ Given that much of our social behaviour is governed by maximizing rewards and minimizing threats, SCARF suggests that individuals resist change due to threats to one or more of the following: status, certainty, autonomy, relatedness, and fairness. The reverse is also true, and these concepts were used as motivators to encourage change.

### 9. Create opportunities for collaboration and distribute them broadly

Medicine often functions within silos. By creating a place in which the silos are merged together through workshops and subcommittee work, new relationships and collaborations for residents and faculty across specialties were formed, connecting over the common interest in medical education. These relationships fostered reciprocal respect and have been useful in promoting the change across PGME. Strong social networks drive change, and all three aspects of social capital development: trust, reciprocity, and recognition, have been fostered with our approach to change.^20^

Allowing RPLs to achieve personal aims, including fulfilling resident research expectations via collaborative medical education research, or engaging in leadership activities, while also achieving the aims of our CBME resident subcommittee, encourages ongoing momentum and motivation for promoting curricular change. Group members are recognized for having useful input and are trusted in their capacity to lead, while also being shown that if they are willing to contribute effort to the project, they will be shown reciprocal effort in providing them with useful opportunities, both clear examples of reciprocity and recognition as a result of our committee.

### 10. Make it sustainable by developing learner capacity

Change is a constant need in medical education, and yet it is fundamentally hard to do. The needs of trainees shift as our understanding and approach to medicine changes with time. Rather than protecting the status quo, we ultimately need to change our approaches to keep up. Maximizing learner engagement in the evolution of medical education develops the collective psychological mindset needed within your educational community to make change easier next time and has the added bonus of developing learners into faculty with experience in medical education and change leadership.

Some changes, like CBME, are more complex than others. These major change projects provide the opportunity to create the infrastructure needed to deal with all types of change in the future. Establishing ongoing opportunities for learners to attend committee meetings, conferences, and workshops, both with time away from clinical duties as well as financial support, is a tangible way to encourage long-term involvement. Shifting the short-term project-oriented CBME resident subcommittee into a ‘Medical Education Resident Committee’ is also useful in ensuring ongoing capacity for resident co-production. Actively changing the structures and conditions of the educational environment to support learner involvement helps learners maintain the power and agency needed to effectively promote change moving forward. Activating learner agency develops the psychological context for change needed to advance resident and faculty co-production as a core component of educational change.^21^

## Conclusion

Transformative change in post-graduate medical education will always be difficult to navigate. Resident trainees are a talented and motivated group of individuals eager to take on leadership roles in the change process, but their efforts can be thwarted if there is a lack of institutional commitment. Our 10 suggestions forfostering resident co-production require traditional leaders to create an environment of trainee empowerment, maximize end-user design thinking, and develop a shared leadership model that goes beyond the conventional. These goals are challenging to achieve, but it has helped us implement our vision of CBME at Queen’s. The specifics of what worked for us may not be a perfect fit in your context, but we believe these principles fornavigating the “black ice” in medical education reform are universal.
